# Modernized architecture may reduce coercion

**DOI:** 10.1192/j.eurpsy.2021.357

**Published:** 2021-08-13

**Authors:** A. Harpøth, H. Kennedy, L. Sørensen

**Affiliations:** 1 Department Of Forensic Psychiatry, AUH, psykiatrien, Aarhus N, Denmark; 2 Department Of Psychiatry, Trinity College- Dublin University, Dublin, Ireland; 3 National Forensic Mental Health Service, Central Mental Hospital Dundrum, Dundrum, Ireland; 4 Department Of Clinical Medicine, Aarhus University- Health, Aarhus N, Denmark

**Keywords:** modernisation, Architecture, Structural milieu, coercion

## Abstract

**Introduction:**

Prevention and treatment of aggression in psychiatric hospitals is achieved through appropriate medical treatment, professional skills, and optimized physical environment and architecture. Coercive measures are used as a last resort. In 2018 Aarhus University Hospital Psychiatry moved from 19th-century asylum buildings to a newly built modern psychiatric hospital. Advances within psychiatric care have rendered the old psychiatric asylum hospitals inadequate for modern treatment of mental disorders.

**Objectives:**

To examine if relocating from a psychiatric hospital, dating from 19th century to a new, modern psychiatric hospital decreased the use of coercive measures.

**Methods:**

This is a retrospective longitudinal study, with a follow-up from 2017 to 2019. We use two designs; 1) a pre-post analysis of the use of coercive measures at Aarhus University Hospital Psychiatry before and after the relocation and 2) a case-control analysis of Aarhus University Hospital Psychiatry and the other psychiatric hospitals in the Central Region. Data will be analyzed in STATA using an interrupted time-series analysis or similar method. Additionally case-mix and sensitivity analysis will be performed.

**Results:**

Preliminary results show a 45% decrease in the total number of coercive measures and a 52% decrease in the use of mechanical restraint. The reduction that may reasonably be attributed to the relocation is still to be determined and will be presented at the congress.

**Conclusions:**

The study may illuminate how future development and planning of psychiatric facilities might improve psychiatric treatment and increase the understanding of how structural changes might contribute the prevention of the use of coercive measures.

**Disclosure:**

No significant relationships.

